# Monitoring Neutron Radiation in Extreme Gamma/X-Ray Radiation Fields

**DOI:** 10.3390/s20030640

**Published:** 2020-01-23

**Authors:** Rusi P. Taleyarkhan

**Affiliations:** College of Engineering, Purdue University, W. Lafayette, IN 47907, USA; rusi@purdue.edu; Tel.:+1-765-313-1876

**Keywords:** extreme, radiation, neutron, photon, photoneutron, gamma, X-ray, TMFD, sensors

## Abstract

The monitoring of neutron radiation in extreme high ≈10^14^ (#/cm^2^-s) neutron/photon fields and at extremely-low (≈10^−3^ #/cm^2^-s) levels poses daunting challenges—important in fields spanning nuclear energy, special nuclear material processing/security, nuclear medicine (e.g., photon-based cancer therapy), and high energy (e.g., dark-matter) research. Variably proportioned (neutron, gammas, X-ray) radiation, spanning 10^−2^–10^9^ eV in energy, is omnipresent from ultra-low (Bq) activity levels (e.g., cosmic rays/ bananas), to extreme high (>10^20^ Bq) levels. E.g., in nuclear reactor cores; in spent nuclear fuel bearing nuclear-explosive-relevant safeguard-sensitive isotopes, such as Pu-239; and in cancer therapy accelerators. The corresponding high to low radiation dose range spans a daunting 10^16^:1 spread—alongside ancillary challenges such as high temperatures, pressure, and humidity. Commonly used neutron sensors get readily saturated even in modest (<1 R/h) photon fields; importantly, they are unable to decipher trace neutron radiation relative to 10^14^ times greater gamma radiation. This paper focuses on sensing ultra-low to high neutron radiation in extremely high photon (gamma-X ray) backgrounds. It summarizes the state-of-art compared to the novel tensioned metastable fluid detector (TMFD) sensor technology, which offers physics-based 100% gamma-blind, high (60–95%) intrinsic efficiency for neutron-alpha-fission detection, even under extreme (≈10^3^ R/h) gamma radiation.

## 1. Introduction

Just what do we mean by “extremely high” radiation fields that pose challenges to the status quo of neutron monitoring? In order to address this question, one must start with a baseline of sorts.

### 1.1. Commonly Encountered Radiation Levels in Everyday Life—Forming a Baseline

We first start with radiation in everyday life for which monitoring is readily accomplished, and then, relative to such radiation levels (comprising varied radiation particles—neutrons, gammas, betas, etc.) we consider radiation levels that are “extreme high”—quantitatively specified, for which state-of-art sensors fail to perform satisfactorily, and for which we describe the novel tensioned metastable fluid detector (TMFD) sensor technology and its validation for applicability in various challenging environments.

Ionizing radiation is encountered in everyday life (e.g., from cosmic rays, terrestrial radiation, radionuclides within our bodies, and from items such as bananas and radon) [1,2,3]. For example, cosmic radiation at sea level constitutes flux (#/cm^2^-s) of electrons (4.5 × 10^−3^), neutrons (6.5 × 10^−3^), and muons (1.9 × 10^−2^), alongside other charged particles. However, the most common form of ionizing radiation is derived from gamma rays (including X-rays) emitted from decay of over 2000 radionuclides and nuclear reactions, such as uranium fission. In daily life, one therefore encounters radiation fluxes in the ≈10^−3^ #/cm^2^-s fluence and radiation intensity fields from background radiation in the 10^−5^ R/h level. The annual dose to humans is well-known to be in the 0.3 Rem/y range from natural sources with variations depending on medical radiation doses (e.g., 1–5 mRem/dental X-ray, to about 1 Rem/CT-scan) [2]. It is also well known that the 90% lethal whole-body acute dose (delivered in minutes to days) is only ≈10^3^ Rem [2,3]. Another metric utilized is the air exposure rate (R/h) for which one normally utilizes 1 R/h as depicting a “high radiation area,” meaning a person working in such a field for only ≈25 h could accumulate the annual maximum (US Nuclear Regulatory Commission regulated) radiation dose limit for radiation workers of ≈25 Rem/y. These metrics provide a baseline of sorts to then consider “extreme” radiation situations. By this measure, the ≈10^3^ Rem lethal dose to humans from extreme in-core (10^10^ R/h) radiation conditions described next would be attained almost instantly, within only ≈10^−7^ h (≈10^−4^ s).

Various types of detectors (e.g., Geiger Muller, ion chambers, Ge-Li) have been developed to detect gammas in low (Bq) to high gamma fields. However, neutron radiation monitoring at both the ultra-low and ultra-high extreme fields is extremely challenging [1,4].

### 1.2. Extreme Radiation Levels and Challenges

With the baseline radiation levels discussion (above), we now turn to what may inarguably constitute extreme radiation environments and challenges for the monitoring of key safety/performance-significant parameters.

#### 1.2.1. Challenge 1: Extreme Radiation within Nuclear Reactor Pressure Vessels

**Figure 1:**
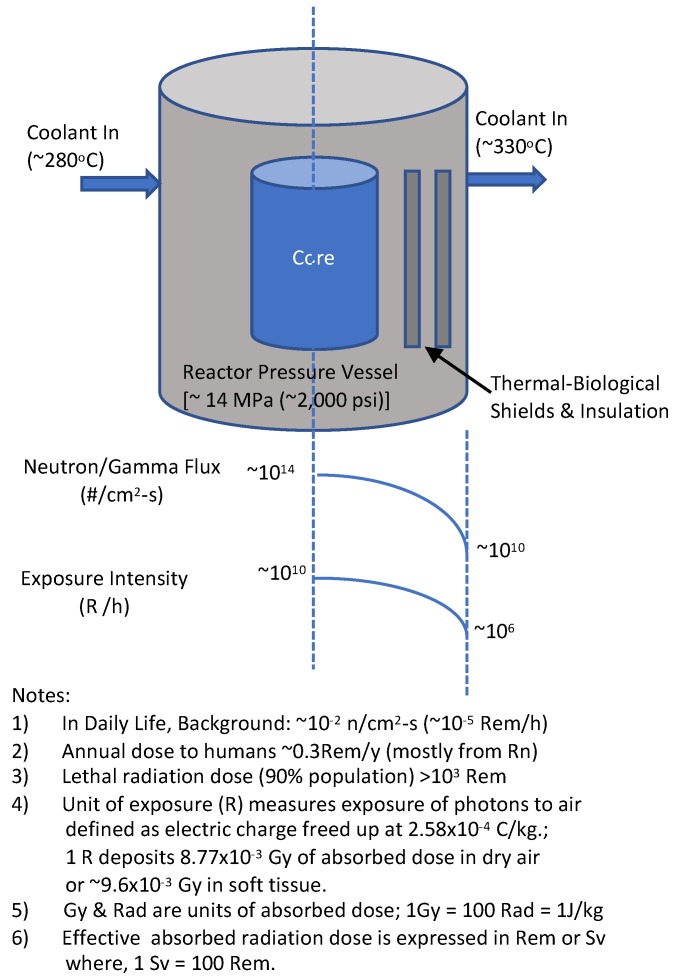
Illustration of extreme radiation conditions in a 3000 MWt nuclear reactor.

Unlike for gamma-beta radiation, neutron radiation is not as commonly encountered; besides constituting the life-blood of a nuclear fission chain reaction, it spontaneously emanates only from decay of very few (≈10–20) radionuclides and constitutes a tell-tale sign for the presence of isotopes of key special nuclear materials (SNMs) undergoing spontaneous fission; e.g., specific isotopes of Pu, U, Cm, and Cf—commonly produced in nuclear fission reactors. In a typical 3000 MWt nuclear light water reactor (LWR), core fission generated neutron and gamma fluxes are comparable (≈10^14^ /cm^2^-s), constituting a radiation environment of ≈10^10^ R/h, as seen from Figure 1 [4]. Very few detectors can function for long within the internals of reactor cores in 10^10^ R/h environments. Only fission chambers with <<1% (spectrum averaged) neutron detection efficiency (basically ion chambers with thin coatings of U-235 or U-238) are amenable to insert for short periods of time to map out the neutron flux within a reactor core; conventional detectors such as He-3 or NE-213 (liquid scintillators), or even CR-39 chips, readily become inoperable in >1 R/h fields. A truly gamma-blind high efficiency spectroscopic neutron detector for this ultra-extreme radiation field within a nuclear fission reactor is yet unavailable. For example, monitoring for neutrons in 10^14^ n/cm^2^-s flux within nuclear reactors is presently feasible only with use of so-called fission chambers which utilize thin (few micron thick) layer deposits of U-235 in gas-filled ion chambers. The high (≈550 b) thermal neutron energy (≈10^−2^ eV) cross-section enables a theoretically high ≈30 cm^−1^ macroscopic cross-section implying a ≈300 µm mean free path; the relatively gamma insensitive neutron detection efficiency for a typical 3 µm thick U-235 layer is only ≈1% (=3/300 × 100) or lower, albeit sufficient for occasional (note: the thin layer can get depleted rapidly) in-core power monitoring neutron fluxes (≈10^14^ n/cm^2^-s). For fast (MeV) energy neutrons, the fission chamber efficiency drops 100 times lower towards 0.01% [1,2,3,4,5]. Therefore, while fission chambers offer a viable option to conduct neutron radiation monitoring in extreme high neutron radiation fields, they are simply impractical for monitoring in majority of everyday situations in environments where the gamma-to-neutron intensity is in the extreme 10^14^:1 range. This is where we focus the discussion in this paper.

#### 1.2.2. Challenge 2: Monitoring for Ultra-Trace Neutron Radiation in Extreme Gamma Background Radiation

**Figure 2:**
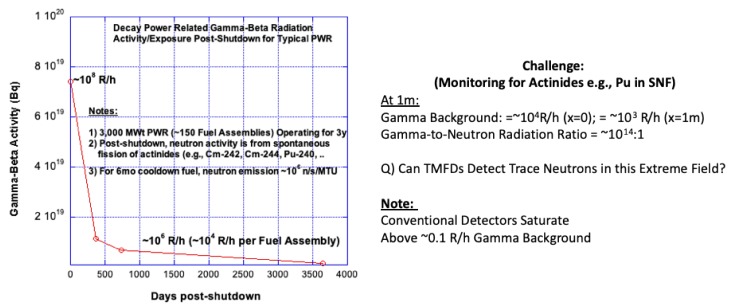
Variation of radiation activity with time for spent nuclear fuel and monitoring challenges in an extreme gamma background.

A separate and unusual security-safeguards challenge arises for extreme radiation fields once a reactor is shut down and the spent nuclear fuel assemblies (SNFAs) are removed for reprocessing. Upon shutdown, the spent nuclear fuel radioactivity level (≈8% of the nominal power) occurs mainly from radioactive decay of fission fragments which are largely gamma-beta radiation emitters, while the relatively small inventory of SNM nuclides decay from spontaneous fission (SF). Figure 2 graphically illustrates the variation [2] of the gamma-beta radiation intensity in Curies (Ci; Note: 1Ci = 3.7 × 10^10^ Bq) due to decay of fission products, e.g., from a 3000 MWt pressurized water reactor (PWR) with time after shutdown. As noted therein, at the end of cycle (EOC), a typical SNF may emit 10^14^ times higher gamma ray intensity (≈10^20^ γ/s/MTU; MTU = metric ton uranium) compared with a relatively miniscule neutron intensity (≈10^6^ n/s/MTU) [6,7]. The radiation field next to a SNF varies with time after shutdown, but even after 1 y of cooldown it can be expected to be above 10^3^–10^4^ R/h. Notably, a 3000 MWt LWR fission reactor can produce ≈1000 kg of Pu, while only ≈8 kg constitutes a threat-level quantity according to the International Atomic Energy Agency [8]. Significant uncertainty can exist in the estimation of Pu buildup during power operation, an aspect which cannot be determined with sufficient confidence using non-destructive assay (NDA) of SNF. Common neutron detectors (e.g., He-3, BF-3) readily get saturated if the background gamma radiation field exceeds ≈1 R/h levels [1]. Therein lies a principle challenge from a safeguards-security perspective—which is to be able to spectroscopically monitor [6,7], successfully, in near real time, the SNM (especially Pu-239, U-235) content from the SNM-specific spectroscopic neutron radiation signature in an extreme (10^14^ times higher) gamma radiation background.

#### 1.2.3. Challenge 3: Monitoring for Ultra-Low/High Neutron Radiation Dose in Extreme “High Energy” X-ray Medical Cancer Therapy Facilities

**Figure 3:**
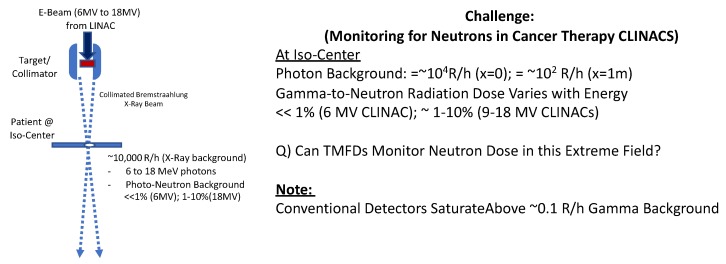
Schematic of extremely high photon fields and low-to-high neutron radiation field monitoring in medical clinical linear accelerators (CLINACs, commonly used worldwide for cancer therapy).

Similar to Challenge 2, another challenge [9] occurs in X-ray photon-based cancer therapy clinical linear accelerator (CLINAC) facilities which employ high energy Bremsstrahlung X-ray generators ranging in end-point energy from 6 MeV towards 18 MeV. Figure 3 schematically depicts a typical CLINAC system and associated challenges involved for extreme radiation field monitoring. The intense photon beams give rise to ≈10^4^ R/h exposure fields, whereas the high energy photons can also readily produce photo-neutrons from the photon–nucleus nuclear reactions with the vast array of element nuclides present (spanning D, C-13, and other shielding elements). Unlike for challenges associated with nuclear fission reactors where the gamma energies are largely below the photo-neutron energy thresholds, for CLINACS which deploy 6–18 MeV X-rays, neutron radiation can become increasingly important as a major source of radiation—for which, at present there is considerable need for novel monitoring technologies (since state-of-art detectors such as CR-39 and TLD type monitors have proven grossly insufficient and/or incapable of separating the neutron signal from the photons).

In CLINAC type conditions, it becomes increasingly important to be able to monitor for the neutron intensity and energy because they can range from relatively insignificant (<0.1% for 6 MV electron accelerators) to significant (≈10% estimated for 18 MV electron accelerators) additional dose to the patient and clinical staff. Large uncertainties can result in the ability to properly monitor for the added radiation dose from neutrons as has been reported [9]. Unlike for gamma (photon) radiation where the radiation effective dose (as expressed commonly in Sv or Rem—with 1 Sv = 100 Rem) to humans is invariant with photon energy, the effective dose from neutrons can vary significantly (by a factor of up to 20) with neutron energy [1]. Not only does one need to properly account for the neutron flux, but, also for the neutron energy spectrum. We note that only ≈10 Sv (1000 Rem) whole body radiation dose constitutes a 90% lethal dose (LD-90) for humans [1,2,3,4,5].

## 2. Materials and Methods—Novel Sensors for Addressing Challenges 1, 2, and 3

As discussed above, for Challenges 1–3, the need exists for the ability to spectroscopically measure neutron fields while remaining relatively blind to extreme background photon (gamma-X ray) radiation. Fortunately, Purdue University has been developing such a sensor technology for gamma-blind detection of neutron, alpha, and fission radiation—one that shows promise for utility in extreme gamma-beta radiation environments. A brief introduction is presented on the underlying science of TMFDs, and that is followed by description of theory and validating experimentation to date which offers evidence for overcoming Challenges 2 and 3.

### 2.1. Introduction to Tensioned Metastable Fluid Detector (TMFD) Sensor Technology

**Figure 4:**
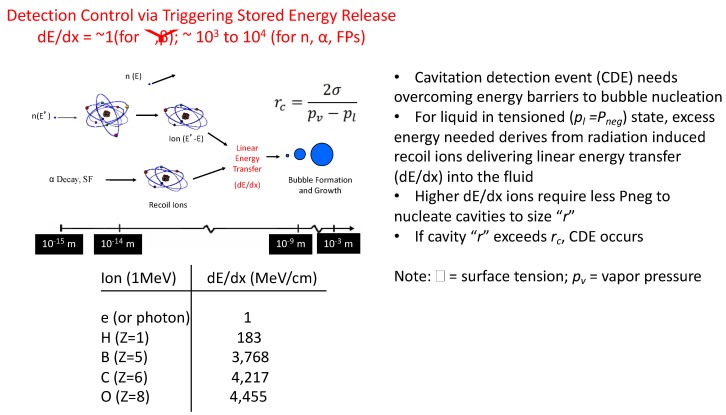
Graphical illustration summarizing key principles in TMFD sensor detection.

Although not widely known, fluids, like solids, can sustain tension (i.e., the intermolecular bonds holding the molecules together can be “stretched” and weakened). Ordinary fluids, such as water, at room temperature can indeed be stretched (i.e., tensioned) to include negative (*P_neg_*) pressures (yes—even below perfect vacuum), as scientifically confirmed only a few decades ago, leading to the novel TMFD sensor class [10,11,12,13,14,15,16]. Briefly, tensioned fluids are in state of metastability; their intermolecular bonds are weakened such that select stimuli types can “poke” holes into them to create transient bubbles that can rapidly (within µs) grow to states that are visible-audible to humans—depicted graphically in Figure 4. Amazingly, conventionally hard to detect sub-atomic neutral particles such as neutrons or ions (tell-tale signatures from U/Pu nuclear fission) can be now detected with unparalleled intrinsic efficiency [12] and spectroscopy even in ultra-low extreme radiation fields (per evidence presented later in this paper). Stimuli types may also include ordinary UV–IR photons. The scientific principles and potential transformational uses have been published elsewhere; e.g., [1,2]. The underlying physics enabling photon-electron blind detection of neutrons and alpha-fission fragments can be appreciated from the inset table of Figure 4, which tabulates the dE/dx (also known as, linear energy transfer or LET) of 1 MeV photons or electrons alongside higher Z ions of TMFD sensor fluid constituent atoms, such as C, F, B, and O. As noted from Figure 4, dE/dx as computed using the well-known SRIM code [17], is 10^3^ times greater for C type high Z ions versus that from photons or light ions like electrons.

#### Underlying Physics-Based Gamma Photon Blind Neutron-Alpha-Fission Detection in TMFDs

Neutrons, being neutral particles, must be detected indirectly; in TMFDs they are detected via the recoiling nuclei of TMFD sensor fluid atoms such as C. It has been found [10] that the recoil energy needs to be above the ≈50–100 keV range for detection in TMFDs. A simple calculation will show that even if a 1 MeV photon interacts head-on with the lightest of atoms (i.e., H), at most the energy imparted to the recoiling proton (A = 1) will be <<100 keV, and furthermore, even smaller for the heavier atoms such as C (A = 12). For example, a 0.67 MeV Cs-137 (a major gamma producing fission product) gamma photon in a head-on collision with a proton (the lightest of atoms in a typical TMFD sensing fluid) can give rise to a recoil proton with a maximum recoil proton energy (Ep) of only ≈0.23 keV (i.e., via combining, E_γ_/c = m_p_ × v_p_, from which, Ep = 0.5 m_p_v_p_^2^). Such low energy recoils simply cannot deposit the required ≈50–100 keV energy deposition within a critical radius, as per nucleation theory, at *P_neg_* states of 10 bar, and hence, the confirmation of the inherent gamma insensitive neutron detection ability of TMFD sensors.

### 2.2. Detection Efficiency of TMFDs versus State-of-Art Sensors

It is important to recognize that conventional neutron detectors (e.g., He-3, BF3, Li) can create signals from both neutron and gamma interactions—especially if the gamma field is high (e.g., >1 R/h). While other detectors exist that claim gamma blindness (e.g., SDDs and CR-39 track-based detectors), these systems exhibit <<1% intrinsic efficiency and can become dysfunctional in moderate neutron fields.

**Figure 5:**
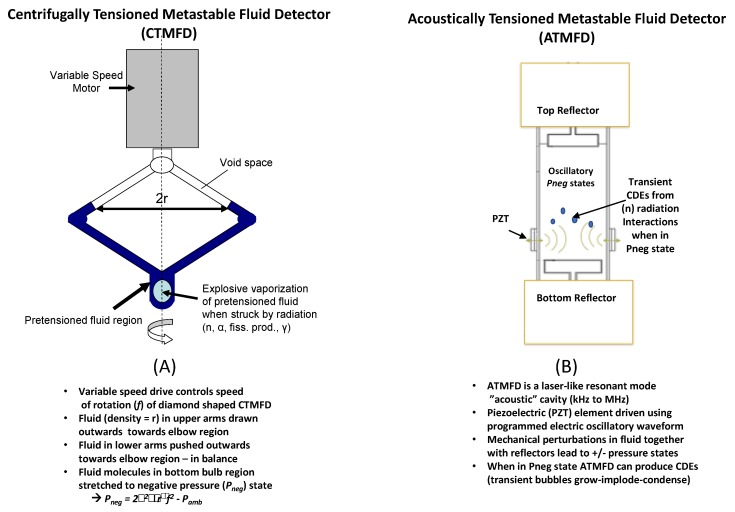
Schematics of CTMFD (**A**) and ATMFD (**B**) sensors and operation basics.

Unlike complex/expensive conventional sensors for radiation-photon detection, which rely on extensive electronic trains, photomultiplier tubes, scintillators, etc., TMFDs are based on intuitive, centrifugal force from common rotary tools and/or resonant mode acoustic vibrations from piezo-electric elements. Two distinct forms of hand portable, table-top systems: C (Centrifugal)-TMFDs and Acoustic (A)-TMFD hand-portable systems, weighing ≈2–3 kg, have been developed for high efficiency spectroscopic neutron-alpha-fission detection and are depicted schematically in Figure 5.

**Figure 6:**
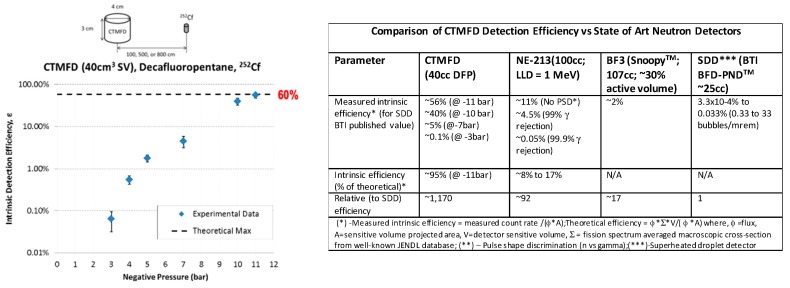
Variation of intrinsic neutron detection efficiency versus *P_neg_* and comparison with state-of-art neutron detectors.

Figure 6 demonstrates high efficiency fission spectrum neutron detection (≈100% of theoretical maximum) intrinsic efficiency which has been found to be ≈75 times higher (per unit volume) than for conventional systems, such as 12 kg, moderated BF_3_ detectors [11,12]. Shown (inset) are comparisons of neutron detection efficiency for TMFDs against the common state-of-art thermal and fast neutron detectors, including superheated drop detectors (SDDs). As noted therein, the per-detector volume-based efficiency of the TMFD can be 1000 times greater than that for SDDs and over 10–100 times higher than other detectors—besides offering the ability to decipher directionality, spectroscopy [13,15], and in gamma blindness even in extreme radiation fields (discussed in Section 3).

## 3. Results—Qualification of TMFDs in Extreme High Gamma Radiation Fields

In order to validate the underlying physics-based ability of TMFD sensors for gamma-blind neutron detection, three sets of experimental efforts have been undertaken so far. These are discussed below:

### 3.1. Gamma Blind Neutron Detection Using a 3 Ci (≈10^11^ γ/s; 5 R/h) Cs-137 Source

**Figure 7:**
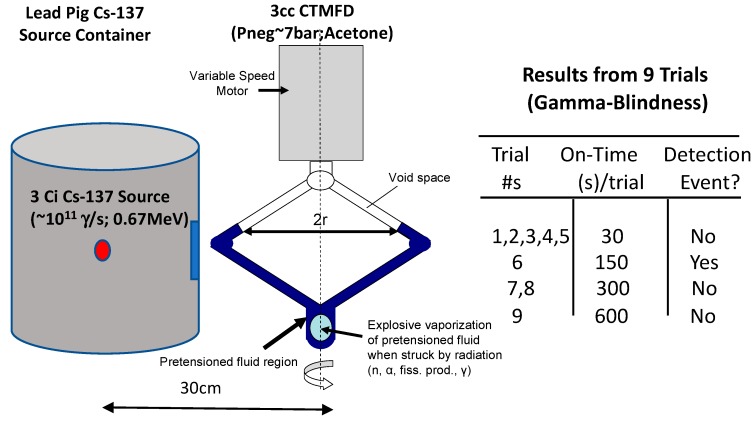
Schematic representation of gamma blind neutron detection using a 3 Ci (Cs-137) gamma source alongside results of various trials.

A CTMFD with a sensitive volume of 3cc using acetone as the sensing fluid was deployed to qualify it for remaining 100% blind to the 0.65 MeV gammas, as seen in Figure 7. The gamma radiation field was monitored as being ≈5 R/h in the vicinity of the CTMFD operating at a *P_ne_*_g_ of ≈7 bar. Experiments were conducted at Rensselaer Polytechnic Institute (RPI) by the author alongside R. Block of RPI [15]. Varying times of on-time for CTMFD exposure were utilized. Only in one of the nine trials (each ranging from 30 s through 600 s as shown in the inset table) did the CTMFD give rise to a single detection event. This was readily associated with detection of the ≈2 × 10^−3^ n/cm^2^-s cosmic neutron flux and was expected.

Notably, in between each of the trials, the CTMFD instantly responded to the presence of a 3Ci PuBe isotope neutron (≈10^7^ n-γ/s) source when brought in the vicinity (with few meters) of the CTMFD. Once the PuBe neutron source was removed to over 10 m away in a shielded room, the CTMFD reliably remained blind to the ≈10^11^ γ/s (monoenergetic pure 0.67 MeV, Cs-137 photon only emitting) source; further details are published elsewhere [14].

These results validated the ability to demonstrate gamma-blind neutron detection in high, multi-R/h pure gamma radiation fields, as may be encountered in the vicinity of strong (yet not instantly lethal) gamma sources that are used in industry for radiation sterilization, radiography, etc.

### 3.2. Qualification in Extreme (≈10^3^ R/h) Multi-Gamma Radiation Fields (Including Photo-Neutrons)

In regard to Challenges 2 and 3 involving extreme (10^3^ to 10^4^ R/h) gamma-photon radiation fields, a 15cc CTMFD using C_5_H_2_F_12_-decafluoropentane (DFP) as the sensing fluid was tested for both gamma-blind neutron detection and photoneutron detection while remaining blind to the extreme (700 R/h) mixed gamma photon field. DFP was chosen in part due to its excellent safety characteristics (i.e., “0,0,0” for fire/reactivity/flammability on the NFPA scale) and its high neutron interaction cross-section.

**Figure 8:**
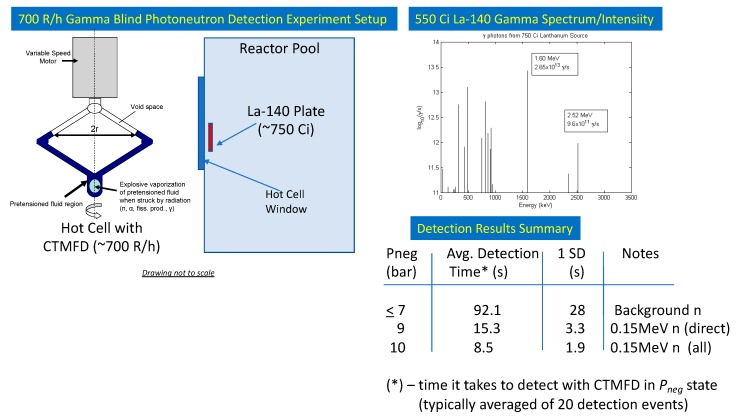
Schematic of experimental setup for qualifying the TMFD for gamma-blindness in 700 R/h multi-gamma energy field along with trace photoneutron spectroscopic detection.

We conducted these qualification experiments [16] at Texas A&M University’s (TAMU’s) 1-MW research reactor facility located in their nuclear science center. In order to produce a pure (multi-monoenergetic energies) gamma field, La-139 was exposed to neutrons in the TRIGA^TM^ reactor to produce a highly activated (≈750 Ci) La-140 source plate. The La-140 was then hung just outside the hot-cell window while remaining in the reactor pool (since it was too “hot” to bring out into the hot cell). Despite the shielding, the gamma exposure at the CTMFD region was estimated to be ≈700 R/h. Figure 8 presents both the photon spectrum with relative intensities for each of the several gamma energies, and the experimental geometry.

As noted in Figure 8 (inset La-140 gamma spectrum [16]), we note the presence of a 2.52 MeV gamma emission (albeit at a 20× lower intensity than for the mainstay 1.6 MeV gamma). Consider that the La-140 source was positioned in the reactor pool water (where about 1:5000 hydrogen atoms will be D atoms). For D atoms the photo-neutron energy threshold is 2.2 MeV, which means one should expect the production of epithermal neutrons of about 0.15 MeV in energy alongside a 0.15 MeV recoil proton; i.e., E_n,p_ (MeV) = (2.52 − 2.2)/2. While the proton will readily get attenuated in the water field, the photo-neutron is then available as a low intensity, low energy particle to escape and interact with the CTMFD, accompanied by photons, within an extreme (700 R/h) gamma field. The challenge now becomes one in which the CTMFD must remain blind to the intense (>10^13^ γ/s) photons while also being able to selectively decipher for the presence of a 100 × lower neutron intensity, and of relatively much lower 0.15 MeV energy too. This challenge was addressed via *P_neg_* threshold based monitoring.

Notably, operating the CTMFD at variable *P_neg_* states, it was found that as the *P_neg_* was sufficiently increased at/above ≈9 bar; the 0.15 MeV neutron and its MCNP transport code estimated intensity could also be detected while the CTMFD was blind to the extreme 10^13^ γ/s gamma radiation field. These results are presented (inset) in Figure 8. The detection time (effectively the inverse of detection rate) is the time it takes for a detection event to occur once the *P_neg_* state has been reached. The second column presents the average value (typically ≈20 detection events).

## 4. Discussion and Conclusions

This paper discusses various scenarios involving challenges pertaining to extreme radiation environments for photon and mixed neutron-photon environments. Challenges are described for neutron radiation detection in extreme radiation environments for conditions within nuclear reactor cores, in the vicinity of spent nuclear fuel assemblies, and in high energy (6– 18 MV) CLINACs employed for cancer therapy worldwide. The novel, TMFD sensor technology attributes and enabling features are summarized.

The manuscript presents evidence of experimentally-derived data for gamma blind neutron detection from two experiments. The first experiment discusses results of qualification of TMFDs for 100% gamma blindness in a pure 0.67 MeV (Cs-137, 3 Ci) gamma field that offered an ≈5 R/h radiation field.

The second experimental campaign involved mixed gamma-neutron fields, as would be anticipated in a medical CLINAC where the photon background is in the 10^4^ R/h range. Experiments were conducted using an irradiated La-140 plate (550 Ci) which caused a 700 R/h photon field. From 0.15 MeV epithermal neutrons produced via photo-neutron interactions of the 2.5 MeV gammas from La-140 with D atoms in water, it was shown that the TMFD remains blind to the 700 R/h photon field by maintaining the *P_neg_* state of the DFP filled CTMFD to below 7 bar. Only when increasing the *P_neg_* towards 9 bar and greater does the CTMFD begin to also provide the ability to monitor the 10x+ lower intensity neutron radiation.

Further assessments are planned to extend the range (and duration) for the applicability of TMFD technology for long-term extreme radiation environments. It is anticipated that at greater exposures, physical phenomena such as radiolysis related false positive detection events may become important to consider for their influences on the detector’s electronic components.

## 5. Patents

Several patents have been filed and awarded pertaining to TMFD sensor technology.
